# High-Frequency Ultrasound Radiomics Combined with Clinical Features for Detecting OMERACT-Defined Metacarpophalangeal Joint Cartilage Damage in Early Rheumatoid Arthritis

**DOI:** 10.3390/diagnostics16121758

**Published:** 2026-06-06

**Authors:** Minghui Yao, Wenxue Li, Yuwei Xin, Diancheng Li, Li Yang, Jia’an Zhu

**Affiliations:** Department of Ultrasound, Peking University People’s Hospital, Beijing 100044, China; 1810301217@bjmu.edu.cn (M.Y.);

**Keywords:** ultrasound radiomics, rheumatoid arthritis, cartilage damage, metacarpophalangeal joint

## Abstract

**Background/Objectives**: The aim of this study was to develop and validate a high-frequency ultrasound radiomics-based model for quantitative assessment of metacarpophalangeal (MCP) joint cartilage damage in early rheumatoid arthritis (RA). **Methods**: 656 MCP joints from 99 early RA patients and 65 healthy controls were prospectively enrolled and graded according to the Outcome Measures in Rheumatology (OMERACT) system. After radiomics feature extraction, five machine learning classifiers were evaluated. Radiomics, clinical, and combined models were constructed and assessed. Radiomics scores were compared among healthy grade 0 joints, early RA grade 0 joints stratified into two risk subgroups, and RA grade ≥ 1 joints. SHapley Additive exPlanations (SHAP) analysis was used for interpretation. **Results**: Eight stable radiomics features were selected. Among classifiers, support vector machine achieved the highest cross-validated performance and was selected as the final radiomics classifier (validation AUC = 0.804). The combined model, integrating radiomics features with age, disease duration, and Disease Activity Score in 28 joints, achieved the best diagnostic performance (AUC = 0.855), significantly outperforming both the radiomics and clinical models. Among OMERACT grade 0 joints, the high-risk subgroup demonstrated elevated radiomics-derived scores. SHAP analysis identified original_shape2D_PerimeterSurfaceRatio as the strongest contributor. **Conclusions**: High-frequency ultrasound radiomics combined with clinical features demonstrated strong performance in detecting MCP joint cartilage damage in early RA and may provide a quantitative extension to conventional semiquantitative assessment.

## 1. Introduction

Rheumatoid arthritis (RA) is a chronic inflammatory autoimmune disease characterized by persistent synovitis and progressive joint destruction, affecting about 0.5–1% of the global population [1]. Damage to articular cartilage is a major contributor to functional impairment, reduced quality of life, and disability in RA patients [2]. Early and accurate detection of cartilage damage is essential for timely intervention and improved outcomes.

High-frequency ultrasound (HFUS) is radiation-free, offers real-time dynamic imaging, and provides high resolution. It is widely used for RA diagnosis and monitoring [3]. Magnetic resonance imaging (MRI) provides excellent soft-tissue contrast and is a reference standard for cartilage evaluation, but its high cost, long examination time, and limited accessibility may constrain its routine application in RA follow-up. Importantly, previous studies have demonstrated that HFUS shows good agreement with MRI for detecting both synovial inflammation and structural damage in MCP joints [4], supporting its use as an alternative. For standardized assessment, the Outcome Measures in Rheumatology (OMERACT) working group developed a validated semi-quantitative cartilage scoring system. This system classifies metacarpophalangeal (MCP) joint cartilage damage into grades 0–2 and is widely adopted in clinical and research settings [5]. However, as a semi-quantitative tool, the system cannot fully capture the continuous nature of cartilage damage. Consequently, joints scored as OMERACT grade 0 may still have subtle structural alterations, leading to underestimation of early cartilage damage.

Radiomics is a quantitative image analysis approach that enables high-throughput extraction of high-dimensional features from medical images [6]. By capturing subtle texture, morphological, and intensity information in regions of interest (ROIs) [7], radiomics has demonstrated significant value for early diagnosis and prognostic stratification of various diseases. In recent years, ultrasound-based radiomics has shown promise in the assessment of RA; however, most studies have focused on synovitis or differential diagnosis [8,9,10], whereas quantitative evaluation of cartilage damage, particularly through integration with clinical parameters, remains limited.

This study aimed to develop a multimodal model integrating ultrasound radiomics with clinical parameters for detecting cartilage damage in early RA. Beyond model development, we further explored whether the radiomics model could identify latent structural heterogeneity within OMERACT grade 0 joints, where conventional semi-quantitative assessment may underestimate early cartilage damage. This approach may offer a reproducible and objective complement to OMERACT ultrasound assessment, supporting earlier and more precise RA cartilage assessment.

## 2. Materials and Methods

### 2.1. Study Population

The single-center, prospective cross-sectional study enrolled patients with RA who attended the Department of Rheumatology at Peking University People’s Hospital and underwent HFUS examinations between September 2023 and January 2026.

The inclusion criteria were as follows: fulfillment of the 2010 American College of Rheumatology/European League Against Rheumatism (ACR/EULAR) classification criteria for RA; age between 18 and 75 years; disease duration of less than 2 years; involvement of at least one second or third MCP joint; and availability of complete baseline clinical and ultrasound imaging data. Early RA was defined as a disease duration of less than 2 years.

The exclusion criteria were as follows: concomitant inflammatory joint diseases (e.g., psoriatic arthritis and gout); history of trauma or surgery; intra-articular corticosteroid injections within the previous 4 weeks; poor ultrasound image quality; severe systemic diseases or malignant tumors; and pregnancy.

Healthy controls were recruited during the same study period. The inclusion criterion was the absence of any history or symptoms of arthritis. Exclusion criteria included any history or current evidence of joint disease and poor ultrasound image quality.

A total of 99 patients with early RA were ultimately included. Bilateral MCP2–3 images were acquired for each patient, resulting in 396 MCP joints for analysis. Patients were randomly divided into a training set and an independent validation set at the patient level in a 7:3 ratio, yielding 69 patients (276 joints) in the training set and 30 patients (120 joints) in the validation set. This patient-level split was used to ensure that joints from the same patient were not allocated to both sets. In addition, 65 healthy controls (260 joints) were included. Baseline clinical data were collected for all RA patients, including the Disease Activity Score in 28 joints (DAS28), erythrocyte sedimentation rate (ESR), C-reactive protein (CRP), anti-cyclic citrullinated peptide antibody (anti-CCP), and rheumatoid factor (RF). DAS28 was calculated using four components: the 28-joint tender joint count (TJC28), the 28-joint swollen joint count (SJC28), the ESR in mm/h, and the general health score (GH) assessed by the patient on a 100 mm visual analogue scale (0 = best, 100 = worst). The DAS28-ESR was computed using the following formula:

$$DAS28=0.56\times \sqrt{TJC28}+0.28\times \sqrt{SJC28}+0.70\times \ln\left(ESR\right)+0.014\times GH$$

### 2.2. Ultrasound Assessment

Ultrasound examinations were performed using a Canon Aplio i800 ultrasound scanner (Canon Inc., Tokyo, Japan) equipped with a 24 MHz high-frequency linear transducer. All examinations were performed by two physicians with over three years’ experience in musculoskeletal ultrasound, following the OMERACT Working Group guidelines. Participants were seated with their hands resting naturally on the examination table, and the fingers were flexed to approximately 60° to fully expose the articular surfaces of the metacarpal heads. The transducer beam was positioned strictly perpendicular to the cartilage surface. Grayscale ultrasound images of the dorsal aspects of bilateral MCP2 and MCP3 joints were acquired along the longitudinal axis of the finger. All images were stored in DICOM format.

**Figure 1:**
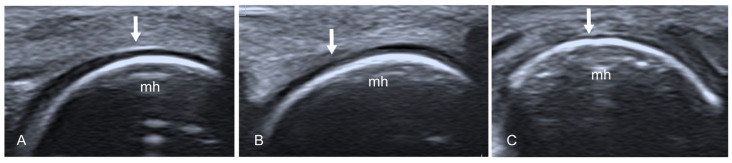
Reference ultrasound images of metacarpophalangeal joint cartilage damage according to the OMERACT scoring system. (**A**) Grade 0: Normal appearance. (**B**) Grade 1: Minimal changes. (**C**) Grade 2: Severe changes. OMERACT, Outcome Measures in Rheumatology; arrows, margin between cartilage and synovium; mh, metacarpal head.

The OMERACT cartilage score was independently assessed by two senior sonographers who were blinded to the patients’ clinical and laboratory data. In cases of disagreement, a third senior expert made the final decision. Cartilage damage was graded as follows: grade 0, normal thickness and echogenicity of hyaline cartilage; grade 1, abnormal cartilage echogenicity (localized increase or decrease), with or without mild thinning; grade 2, marked reduction in cartilage thickness or complete loss of cartilage (Figure 1).

### 2.3. Radiomic Features Extraction

#### 2.3.1. Image Segmentation

All ultrasound images were manually segmented by one experienced sonographer, who was blinded to clinical and laboratory data, using 3D Slicer software (version 5.10.0). The ROI was delineated on the longitudinal dorsal grayscale image of each MCP joint, encompassing the visible hyaline cartilage over the metacarpal head. The superficial boundary was defined by the cartilage–synovial interface, and the deep boundary was defined by the cartilage–bone interface (identified as the hyperechoic cortical surface). The ROI included only the visible cartilage layer and excluded adjacent synovium, cortical bone, joint fluid, and regions affected by shadowing or artifacts. To assess reproducibility, 50 joint images were randomly selected and independently segmented by two sonographers. Dice similarity coefficient and intraclass correlation coefficient (ICC) were calculated.

#### 2.3.2. Feature Extraction

Before radiomics feature extraction, ultrasound images and their corresponding masks were resampled to a uniform spacing of 0.0154 × 0.0154 mm using B-spline interpolation. Intensity normalization was performed using z-score standardization, with the normalized values subsequently scaled to a range of 0–100. Gray-level discretization was conducted using a fixed bin width of 16. As all images represented single two-dimensional cross-sectional slices, feature extraction was restricted to the two-dimensional mode throughout.

Radiomics features were extracted from each ROI using the PyRadiomics package (version 3.1.0) in Python (version 3.9.25), in accordance with the Image Biomarker Standardization Initiative (IBSI) recommendations [11]. A total of 939 features were initially extracted, including shape features, gray-level co-occurrence matrix (GLCM) features, gray-level run-length matrix (GLRLM) features, gray-level size zone matrix (GLSZM) features, gray-level dependence matrix (GLDM) features, and features derived from image transformations, including wavelet decomposition (LL, LH, HL, HH), gradient, logarithm, exponential, and square filters.

#### 2.3.3. Feature Selection

Feature selection was performed exclusively within the RA training set to avoid data leakage. The outcome was dichotomized as OMERACT grade 0 versus OMERACT grade ≥1. Repeated stratified 5-fold cross-validation was used for feature selection. In each fold, features with unstable repeatability (ICC < 0.75) were excluded. Low-variance features were removed using a variance threshold of 0.01. Redundant features with an absolute correlation coefficient greater than 0.90 were further excluded, with priority given to features showing stronger correlation with the outcome variable. Univariate screening was then performed using the Mann–Whitney U test, and features with *p* < 0.05 were retained. The remaining features were standardized using Z-score normalization, and least absolute shrinkage and selection operator (LASSO) regression with 10-fold cross-validation was applied. The optimal regularization parameter λ was determined using the minimum error criterion, and features with non-zero coefficients were retained. This process was repeated independently in each fold, and the selection frequency of each feature across all repeated cross-validation runs was calculated. Features with a selection frequency of at least 0.20 were ultimately selected to construct a final stable feature set for subsequent model construction.

#### 2.3.4. Construction of Radiomics Machine Learning Models

Based on the final stable feature set, radiomics classification models were constructed. To compare the performance of different classifiers under identical feature inputs, five machine learning algorithms were trained and evaluated: Random Forest, Gradient Boosting (GB), Logistic Regression (LR), k-Nearest Neighbor (KNN), and Support Vector Machine (SVM). All models were fitted in the training set and evaluated in the validation set. The classification threshold was determined using the Youden index. Machine learning analyses were implemented using scikit-learn (version 1.6.1).

Receiver operating characteristic (ROC) curves for each model were generated for both the training and validation sets. Model performance was assessed using the area under the receiver operating characteristic curve (AUC), accuracy, sensitivity, specificity, and F1-score. The 95% confidence intervals of AUCs were estimated using Bootstrap resampling (2000 iterations). Statistical comparisons of AUC differences between models were performed using the DeLong test.

Hyperparameter optimization was performed independently for each classifier using a two-stage procedure. In the first stage, RandomizedSearchCV was applied over a broad, pre-specified hyperparameter search space to efficiently explore a wide range of parameter configurations. In the second stage, GridSearchCV was applied within a refined search space centered on the promising regions identified in the first stage. Both stages employed 5-fold stratified cross-validation with the AUC as the optimization criterion. Class-weighted settings were included in the hyperparameter grids to account for class imbalance. The final hyperparameters and cross-validation results are summarized in Supplementary Table S1.

The candidate models were initially ranked according to the mean cross-validated AUC (CV-AUC) within the training cohort. When models showed comparable AUCs, F1-score and the training–validation performance gap were additionally considered to select models with better balanced classification performance and generalizability. The machine learning model with the best performance was selected as the method for subsequent modeling.

Furthermore, the SHapley Additive exPlanations (SHAP; version 0.49.1) analysis was performed to interpret the final machine learning model. SHAP summary bar plots and beeswarm plots were generated to quantify the relative contribution of each feature to the model’s prediction.

To explore whether the radiomics-derived score might reflect subclinical cartilage changes in very early RA patients without conventional structural damage, an exploratory analysis was conducted within patients with duration ≤ 3 months. Within this early RA grade 0 cohort, joints were stratified according to a clinically high-risk inflammatory phenotype, defined as seropositivity (anti-CCP > 20 U/mL or RF > 20 IU/mL) together with moderate-to-high disease activity (DAS28 > 3.2), as seropositivity and high disease activity are recognized poor prognostic factors [12]; the phenotype-negative group included joints from patients not simultaneously meeting both criteria. Age-adjusted radiomics-derived scores were compared among four groups: grade 0 joints from healthy controls, phenotype-negative early RA grade 0 joints, phenotype-positive early RA grade 0 joints, and grade ≥ 1 joints from RA patients. Overall differences among the four groups were assessed using the Kruskal–Wallis test. Pairwise comparisons between groups were conducted using the Mann–Whitney U test. The distributions of radiomics-derived scores across the four groups were visualized using violin plots.

#### 2.3.5. Construction and Comparison of Radiomics, Clinical, and Combined Models

To further evaluate the combined value of radiomic features and clinical information, this study constructed the following three types of models within the RA group. The clinical model was constructed using clinical variables including age, disease duration, and DAS28. The radiomics model was constructed using the final stable radiomics feature set. The combined model incorporated the radiomics-derived score together with the clinical variables to construct a multimodal model. Given the limited number of input variables, logistic regression was selected as the classifier for both the clinical and combined models. The three models were evaluated in the independent validation set. ROC curves were plotted, and AUCs were compared using the DeLong test. To account for within-patient correlation among multiple joints, patient-clustered bootstrap resampling was additionally performed as a sensitivity analysis.

#### 2.3.6. Statistical Analysis

All statistical analyses and visualizations were performed using Python (version 3.9.25) and R (version 4.3.2). Continuous variables were tested for normality using the Shapiro–Wilk test. Normally distributed variables were presented as mean ± standard deviation and compared using the independent-samples *t*-test. Non-normally distributed variables were presented as median and interquartile range, and compared using the Mann–Whitney U test. Categorical variables were presented as frequencies and proportions (%) and compared using the chi-square test or Fisher’s exact test. For multiple pairwise comparisons, Bonferroni correction was applied where appropriate. All statistical tests were two-sided, with *p* < 0.05 considered statistically significant.

## 3. Results

### 3.1. Baseline Characteristics

A total of 99 patients with early RA and 65 healthy controls, with a total of 656 MCP joints, were included in the analysis. As shown in Table 1, there were no significant differences between the RA group and the healthy control group in terms of age or sex distribution. In contrast, regarding the distribution of OMERACT cartilage grades, cartilage damage was significantly more prevalent in the RA group than in the healthy control group.

### 3.2. Radiomics Feature Extraction and Selection

A total of 939 radiomic features were initially extracted from the MCP ultrasound images. Eight stable features were ultimately retained and their frequencies are listed in Table 2. Inter-observer reproducibility analysis demonstrated excellent agreement, with a Dice coefficient of 0.88 ± 0.04.

### 3.3. Model Performance Comparison

**Figure 2:**
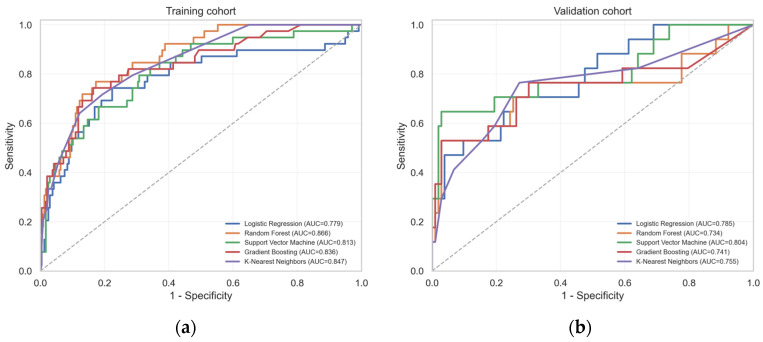
Receiver operating characteristic (ROC) curves of five machine learning models for radiomics-based classification in the (**a**) training and (**b**) validation cohort.

Using the selected radiomics feature set as input, the diagnostic performance of five machine learning algorithms (LR, random forest, SVM, GB, and KNN) was systematically evaluated after hyperparameter tuning (Table 3 and Figure 2). Among the candidate radiomics classifiers, SVM achieved the highest mean CV-AUC (0.796). In the validation cohort, SVM achieved a validation AUC of 0.804 and showed comparable performance to LR (*p* = 0.631) and KNN (*p* = 0.074). SVM demonstrated significantly better discrimination than random forest (*p* < 0.001) and GB (*p* = 0.027). In addition, SVM demonstrated stable performance across the training and validation cohorts and showed favorable validation accuracy (81.7%) and specificity (84.5%). Given its highest CV-AUC and stable generalization performance across cohorts, SVM was selected as the final radiomics classifier.

**Figure 3:**
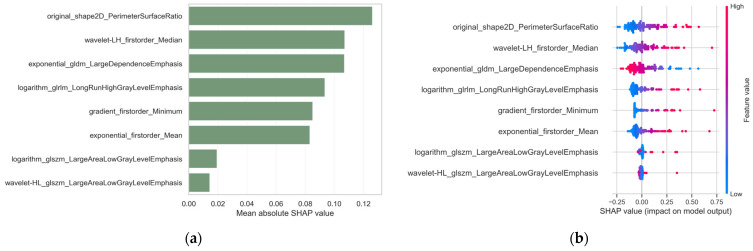
SHAP-based global interpretability analysis of the radiomics model. (**a**) SHAP feature importance bar plot ranking variables by mean absolute SHAP values; (**b**) SHAP beeswarm plot showing the distribution of SHAP values per feature across all samples; color indicates feature value (red: high, blue: low). SHAP: SHapley Additive exPlanations.

To improve model interpretability and quantify the contribution of individual radiomics features to model predictions, SHAP-based global interpretability analysis was performed. SHAP analysis identified original_shape2D_PerimeterSurfaceRatio as the most influential feature, followed by wavelet-LH_firstorder_Median, exponential_gldm_LargeDependenceEmphasis, and logarithm_glrlm_LongRunHighGrayLevelEmphasis. The SHAP beeswarm plot further illustrated the direction and magnitude of each radiomics feature’s contribution to the predicted probability (Figure 3).

**Figure 4:**
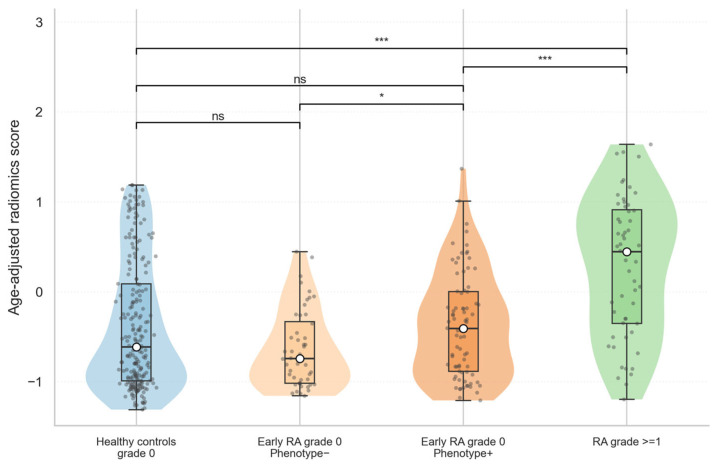
Violin plots showing the distribution of age-adjusted radiomics scores across four groups. Phenotype+ was defined as seropositivity (anti-CCP > 20 U/mL or RF > 20 IU/mL) with DAS28 > 3.2 among RA grade 0 joints with disease duration ≤ 3 months. Phenotype− indicates early RA grade 0 joints not simultaneously meeting both criteria. ns, not significant; * *p* < 0.05; *** *p* < 0.001.

Age-adjusted radiomics-derived scores differed significantly among the four groups (Kruskal–Wallis test, H = 42.09, *p* < 0.001). The median radiomics-derived score (Rad-score) was −0.613 [IQR: −0.992 to 0.088] in healthy control grade 0 joints, −0.743 [−1.016 to −0.331] in phenotype-negative early RA grade 0 joints, −0.408 [−0.886 to 0.001] in phenotype-positive early RA grade 0 joints, and 0.449 [−0.351 to 0.914] in RA joints with grade ≥1 damage. No significant difference was observed between healthy control grade 0 joints and phenotype-negative early RA grade 0 joints (*p* = 0.111). However, within RA grade 0 joints with disease duration ≤3 months, the phenotype-positive subgroup showed significantly higher Rad-scores than the phenotype-negative subgroup (*p* = 0.016). This subgroup showed Rad-scores comparable to those of healthy control grade 0 joints. RA joints with grade ≥ 1 damage showed significantly higher Rad-scores than all other groups (all *p* < 0.001) (Figure 4).

### 3.4. Comparison of Radiomics, Clinical and Combined Models

**Figure 5:**
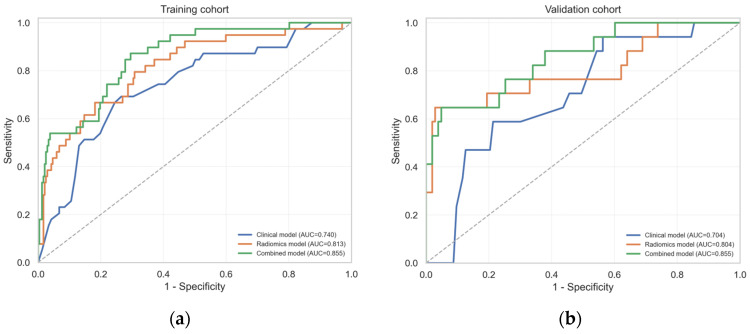
Comparison of the diagnostic performance of the clinical, radiomics, and combined models. Receiver operating characteristic (ROC) curves in (**a**) the training cohort and (**b**) the validation cohort.

**Figure 6:**
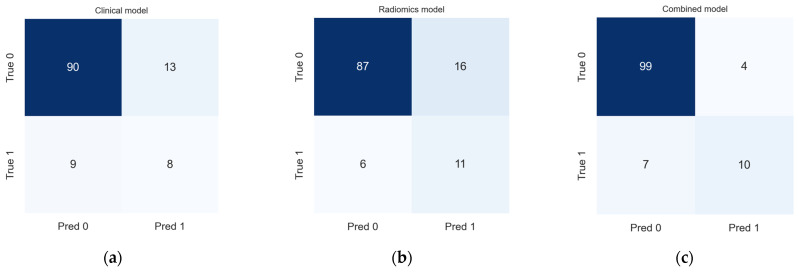
Confusion matrices of the (**a**) clinical, (**b**) radiomics, and (**c**) combined model in the validation cohort.

The diagnostic performance of the clinical, radiomics, and combined models was compared. The clinical model, based solely on clinical variables, showed limited discriminative ability, with an AUC of 0.704 (95% CI: 0.568–0.817), a sensitivity of 47.1%, and a specificity of 87.4%. The radiomics model achieved improved performance, with an AUC of 0.804 (95% CI: 0.660–0.924), a sensitivity of 64.7%, and a specificity of 84.5%. The combined model, integrating the radiomics-derived score with clinical variables, demonstrated the best performance, with an AUC of 0.855 (95% CI: 0.760–0.941), a sensitivity of 58.8%, and a specificity of 96.1%. The DeLong test showed that the combined model significantly outperformed the clinical model (*p* = 0.032) and the radiomics model (*p* = 0.039) (Figure 5). Patient-clustered bootstrap analysis yielded similar results, with the combined model maintaining the highest discriminative performance (clustered AUC = 0.825), though the difference did not reach statistical significance compared with the radiomics model (AUC = 0.782). Confusion matrix analysis further demonstrated that the combined model achieved the highest specificity while maintaining acceptable sensitivity, compared with the clinical and radiomics models (Figure 6). The combined model achieved the lowest Brier score (0.078), compared with the clinical model (0.116) and the radiomics model (0.085), indicating improved probabilistic prediction accuracy.

## 4. Discussion

The OMERACT ultrasound cartilage scoring system is widely used for the semi-quantitative assessment of articular cartilage damage in RA due to its good sensitivity and feasibility [5]. Although it is easy to apply in clinical practice, the system classifies all grade 0 joints as structurally normal and does not provide quantitative discrimination between grade 0 and grade 1. In fact, from a histopathological perspective, cartilage damage in RA is a continuous process. Under persistent synovial inflammation and pannus invasion, early changes, such as proteoglycan loss and disorganization of collagen fibers may occur before macroscopically visible physical thinning of the cartilage [13,14]. Consequently, OMERACT grade 0 may not necessarily represent a biologically homogeneous state, and may include joints at the threshold of structural damage. To our knowledge, few studies have specifically investigated ultrasound radiomics of MCP joint cartilage in early RA using OMERACT-defined cartilage grades as standardized labels to develop a quantitative extension of semi-quantitative ultrasound assessment. Clinically, such an approach may complement conventional ultrasound assessment by enabling more objective evaluation of subtle cartilage alterations and by identifying potentially high-risk grade 0 joints in early RA.

The principal finding of this study is the successful development of a radiomics-based model for detecting cartilage damage in early RA patients, which demonstrated the ability to capture subtle structural abnormalities. Previous ultrasound radiomics studies in RA patients have more commonly focused on synovitis or bone erosion [8,10], whereas cartilage radiomics studies have mainly focused on its degeneration in osteoarthritis [15]. By specifically targeting MCP cartilage in early RA, our study provides a new imaging perspective for the assessment of early structural cartilage involvement. Although no significant difference was observed between RA grade 0 joints and healthy control grade 0 joints at the group level, the distribution of radiomics-derived scores suggested substantial heterogeneity within RA grade 0 joints. This concept was further supported by the exploratory subgroup analysis, in which phenotype-positive early RA grade 0 joints, defined by seropositivity and moderate-to-high disease activity within 3 months of disease duration, demonstrated higher age-adjusted radiomics-derived scores than phenotype-negative early RA grade 0 joints. Because these factors are considered poor prognostic indicators associated with disease progression and joint damage in RA [12], the elevated radiomics-derived scores observed in this subgroup may represent a preliminary signal of potential subclinical structural alterations and should therefore be interpreted cautiously. Future studies with larger cohorts and follow-up are needed to determine whether these grade 0 joints with higher radiomics scores are at increased risk of progression.

Another important finding of this study is that the combined model integrating ultrasound radiomics with clinical features demonstrated better diagnostic performance than either single-modality model. Clinical variables such as DAS28 and disease duration reflect the patient’s overall disease status, but they cannot fully capture the heterogeneity of joint-level cartilage damage. Age is also an important factor, consistent with the well-established age-dependent progression of cartilage damage in RA and the compounding effect of aging on joint structural integrity [16]. Conversely, radiomics features provide quantitative information on local cartilage morphology and texture, but do not directly reflect systemic disease activity. In the present study, the combined model achieved the highest validation AUC (0.855, 95% CI: 0.760–0.941), outperforming both the clinical model (AUC = 0.704, 95% CI: 0.568–0.817) and the radiomics model (AUC = 0.804, 95% CI: 0.660–0.924). Patient-level clustered bootstrap analysis showed a similar performance trend, though the differences did not reach statistical significance. These findings suggest that clinical and radiomics features provide complementary information and that their integration may better characterize the complex mechanisms underlying early cartilage damage in RA. Similar advantages of combining radiomics features with clinical variables have been reported in previous radiomics studies, supporting the value of multimodal prediction models in medical imaging [17,18].

In the algorithm comparison, different classifiers showed distinct performance patterns. Among the evaluated machine learning models, SVM achieved the highest repeated cross-validated AUC and maintained relatively stable performance across the training and validation cohorts. In contrast, tree-based ensemble methods such as random forest and GB showed higher AUCs in the training cohort but lower performance in the validation cohort, suggesting potential overfitting [19]. Although complex machine learning algorithms can model nonlinear relationships, they may require larger datasets to generalize reliably. In contrast, SVM is suited for moderate-sized and high-dimensional datasets, by maximizing the decision margin between classes, a principle that inherently constrains model complexity and limits overfitting. Rather than claiming universal superiority, these results indicate that SVM achieved the best balance between discriminative performance and generalization in this specific dataset.

In addition, the relatively low proportion of structurally damaged joints may have influenced the stability of threshold-dependent metrics, including sensitivity, specificity, and F1-score. To mitigate this issue, class weights were included in the hyperparameter tuning grid, and stratified cross-validation was used during model development. Bootstrap confidence intervals and confusion matrices were reported to provide a more comprehensive assessment of model uncertainty and classification behavior. Nevertheless, class imbalance remains an inherent challenge in early RA imaging studies and may partly explain the variability in sensitivity and specificity across different machine learning algorithms.

The SHAP analysis further improved the interpretability of the radiomics model by quantifying the relative contributions of individual features. The most influential feature was original_shape2D_PerimeterSurfaceRatio, followed by exponential_gldm_LargeDependenceEmphasis, wavelet-LH_firstorder_Median, and logarithm_glrlm_LongRunHighGrayLevelEmphasis. From a pathological perspective, these features may reflect different aspects of cartilage microstructural alteration [20]. Original_shape2D_PerimeterSurfaceRatio is a shape-related feature that may reflect boundary complexity or surface irregularity, which may be associated with early roughening or focal defects of the cartilage surface. Texture-related features, such as logarithm_glrlm_LongRunHighGrayLevelEmphasis and wavelet-LH_firstorder_Median, may reflect changes in signal intensity distribution and textural heterogeneity of the cartilage matrix [21]. In contrast, exponential_gldm_LargeDependenceEmphasis showed an inverse association with cartilage damage, suggesting that loss of homogeneous local texture patterns may be related to matrix disruption. Overall, these findings indicate that ultrasound radiomics may capture subvisual changes in cartilage texture and morphology and share a similar pathological rationale with changes in textural features observed in previous OA cartilage radiomics studies [22,23].

This study has several limitations. First, this was a cross-sectional single-center study, and all ultrasound images were acquired using a single ultrasound platform, which may limit the generalizability of the proposed radiomics model. External validation using multicenter datasets and different ultrasound platforms is therefore needed. Second, the relatively limited sample size and the low proportion of structurally damaged joints may have compromised the model’s stability. Third, the absence of follow-up precluded evaluation of whether elevated radiomics-derived scores in grade 0 joints are associated with subsequent structural progression or treatment response. Finally, MRI was not included as a reference modality, and future studies integrating MRI-based structural evaluation may help further validate the pathological significance of ultrasound radiomics-derived cartilage alterations.

## 5. Conclusions

This study demonstrated that a combined model integrating high-frequency ultrasound radiomics with clinical features (age, disease duration, and DAS28) achieved favorable diagnostic performance for detecting metacarpophalangeal joint cartilage damage in early RA. The present study further supports the role of ultrasound radiomics in RA by providing a quantitative extension to conventional semiquantitative cartilage assessment and by revealing potential structural heterogeneity within OMERACT grade 0 joints.

## Figures and Tables

**Table 1:** Baseline demographics and disease characteristics of patients with RA and HCs.

Characteristics	RA Patients	HCs	*p*-Value
Number	99	65	
Age, years, mean (SD)	48.8 (13.8)	44.9 (11.8)	0.052
Female, *n* (%)	76 (76.8%)	55 (84.6%)	0.239
Disease duration, months, median (IQR)	6.0 (2.5–13.5)	-	-
CRP, mg/L, median (IQR)	0.8 (0.0–8.6)	-	-
ESR, mm/h, median (IQR)	16.0 (8.0–28.5)	-	-
Anti-CCP, IU/mL, median (IQR)	236.5 (101.7–304.0)	-	-
RF, IU/mL, median (IQR)	53.7 (16.3–178.4)	-	-
DAS 28-ESR, median (IQR)	4.07 (3.17–5.24)	-	-
Joints grade = 0, *n* (%)	340 (85.9%)	248 (95.4%)	<0.001
Joints grade = 1, *n* (%)	48 (12.1%)	9 (3.5%)	<0.001
Joints grade = 2, *n* (%)	8 (2.0%)	3 (1.2%)	0.54

RA: rheumatoid arthritis; HCs: healthy controls; SD: standard deviation; RF: rheumatoid factor; anti-CCP: anti-citrullinated peptide antibodies; IQR: interquartile range; ESR: erythrocyte sedimentation rate; CRP: C-reactive protein; DAS28-ESR: disease activity score in 28 joints based on ESR.

**Table 2:** Stable radiomics features and their selection frequencies.

Features	Frequency
logarithm_glrlm_LongRunHighGrayLevelEmphasis	0.84
original_shape2D_PerimeterSurfaceRatio	0.64
logarithm_glszm_LargeAreaLowGrayLevelEmphasis	0.60
wavelet-LH_firstorder_Median	0.58
gradient_firstorder_Minimum	0.52
wavelet-HL_glszm_LargeAreaLowGrayLevelEmphasis	0.48
exponential_gldm_LargeDependenceEmphasis	0.34
exponential_firstorder_Mean	0.20

GLRLM, gray-level run length matrix; GLSZM, gray-level size zone matrix; GLDM, gray-level dependence matrix. Selection frequency indicates the proportion of times each feature was selected across repeated cross-validation during the feature selection process.

**Table 3:** Performance comparison of machine learning models in the training and validation cohorts.

Model	Set	AUC [95% CI]	Accuracy, %	Sensitivity, %	Specificity, %	F1-Score, %
Logistic Regression	Train	0.779 [0.680–0.868]	82.6	59.0	86.5	48.9
	Validation	0.785 [0.670–0.900]	79.2	52.9	83.5	41.9
Random Forest	Train	0.866 [0.807–0.917]	84.8	71.8	86.9	57.1
	Validation	0.734 [0.593–0.894]	79.2	58.8	82.5	44.4
Support Vector Machine	Train	0.813 [0.735–0.880]	81.9	61.5	85.2	49.0
	Validation	0.804 [0.663–0.929]	81.7	64.7	84.5	50.0
Gradient Boosting	Train	0.836 [0.768–0.901]	84.4	69.2	86.9	55.7
	Validation	0.741 [0.588–0.890]	79.2	58.8	82.5	44.4
K-Nearest Neighbors	Train	0.847 [0.796–0.910]	84.4	64.1	87.8	53.8
	Validation	0.755 [0.613–0.890]	80.0	52.9	84.5	42.9

AUC, area under the receiver operating characteristic curve; CI, confidence interval.

## Data Availability

The original contributions presented in this study are included in the article/Supplementary Material. Further inquiries can be directed to the corresponding author.

## Supplementary Materials

The following supporting information can be downloaded at: https://www.mdpi.com/article/10.3390/diagnostics16121758/s1, Table S1: Hyperparameter search space and optimization results.

## Abbreviations

The following abbreviations are used in this manuscript:

RA	Rheumatoid arthritis
HFUS	High-frequency ultrasound
OMERACT	Outcome Measures in Rheumatology
MCP	Metacarpophalangeal
ROIs	Regions of interest
ACR/EULAR	American College of Rheumatology/European League Against Rheumatism
anti-CCP	Anti-cyclic citrullinated peptide antibody
RF	Rheumatoid factor
DAS28	Disease Activity Score in 28 joints
TJC28	The 28-joint tender joint count
SJC28	The 28-joint swollen joint count
ESR	Erythrocyte sedimentation rate
GH	General health score
CRP	C-reactive protein
ICC	Intraclass correlation coefficient
IBSI	Image Biomarker Standardization Initiative
LASSO	Least absolute shrinkage and selection operator
GLCM	Gray-level co-occurrence matrix
GLRLM	Gray-level run-length matrix
GLSZM	Gray-level size zone matrix
GLDM	Gray-level dependence matrix
GB	Gradient Boosting
LR	Logistic Regression
KNN	k-Nearest Neighbour
SVM	Support Vector Machine
SHAP	SHapley Additive exPlanations
ROC	Receiver operating characteristic curve
AUC	Area under the receiver operating characteristic curve
CV-AUC	Cross-validated area under the receiver operating characteristic curve
Rad-score	Radiomics-derived score

## References

[1] Smolen J.S., Aletaha D., McInnes I.B. (2016). Rheumatoid arthritis. Lancet.

[2] Firestein G.S., McInnes I.B. (2017). Immunopathogenesis of Rheumatoid Arthritis. Immunity.

[3] Salaffi F., Gutierrez M., Carotti M. (2014). Ultrasound versus conventional radiography in the assessment of bone erosions in rheumatoid arthritis. Clin. Exp. Rheumatol..

[4] Szkudlarek M., Klarlund M., Narvestad E., Court-Payen M., Strandberg C., Jensen K.E., Thomsen H.S., Ostergaard M. (2006). Ultrasonography of the metacarpophalangeal and proximal interphalangeal joints in rheumatoid arthritis: A comparison with magnetic resonance imaging, conventional radiography and clinical examination. Arthritis Res. Ther..

[5] Mandl P., Studenic P., Filippucci E., Bachta A., Backhaus M., Bong D., Bruyn G.A.W., Collado P., Damjanov N., Dejaco C. (2019). Development of semiquantitative ultrasound scoring system to assess cartilage in rheumatoid arthritis. Rheumatology.

[6] Lambin P., Rios-Velazquez E., Leijenaar R., Carvalho S., van Stiphout R.G., Granton P., Zegers C.M., Gillies R., Boellard R., Dekker A. (2012). Radiomics: Extracting more information from medical images using advanced feature analysis. Eur. J. Cancer.

[7] Gillies R.J., Kinahan P.E., Hricak H. (2016). Radiomics: Images Are More than Pictures, They Are Data. Radiology.

[8] Yan L., Xu J., Ye X., Lin M., Gong Y., Fang Y., Chen S. (2025). Development and validation of ultrasound-based radiomics deep learning model to identify bone erosion in rheumatoid arthritis. Clin. Rheumatol..

[9] Bilgin E. (2025). Current application, possibilities, and challenges of artificial intelligence in the management of rheumatoid arthritis, axial spondyloarthritis, and psoriatic arthritis. Ther. Adv. Musculoskelet. Dis..

[10] Tian H., Liu J., Li S., Yang T. (2026). Development and evaluation of multimodal ultrasound radiomics models for predicting active inflammation in rheumatoid arthritis. Skelet. Radiol..

[11] Zwanenburg A., Vallieres M., Abdalah M.A., Aerts H., Andrearczyk V., Apte A., Ashrafinia S., Bakas S., Beukinga R.J., Boellaard R. (2020). The Image Biomarker Standardization Initiative: Standardized Quantitative Radiomics for High-Throughput Image-based Phenotyping. Radiology.

[12] Smolen J.S., Edwards C.J., Konzett V., Laskou F., Aletaha D., Caporali R., Dorner T., Hyrich K.L., Mateus E., Pope J.E. (2026). EULAR recommendations for the management of rheumatoid arthritis with synthetic and biologic disease-modifying antirheumatic drugs: 2025 update. Ann. Rheum. Dis..

[13] Goldring S.R. (2003). Pathogenesis of bone and cartilage destruction in rheumatoid arthritis. Rheumatology.

[14] Ostrowska M., Maslinski W., Prochorec-Sobieszek M., Nieciecki M., Sudol-Szopinska I. (2018). Cartilage and bone damage in rheumatoid arthritis. Reumatology.

[15] Kiso T., Okada Y., Kawata S., Shichiji K., Okumura E., Hatsumi N., Matsuura R., Kaminaga M., Kuwano H., Okumura E. (2025). Ultrasound-based radiomics and machine learning for enhanced diagnosis of knee osteoarthritis: Evaluation of diagnostic accuracy, sensitivity, specificity, and predictive value. Eur. J. Radiol. Open.

[16] Martin J.A., Buckwalter J.A. (2002). Aging, articular cartilage chondrocyte senescence and osteoarthritis. Biogerontology.

[17] Flaiban E., Orhan K., Goncalves B.C., Lopes S., Costa A.L.F. (2025). Radiomics in Action: Multimodal Synergies for Imaging Biomarkers. Bioengineering.

[18] Zhao J., Sun Z., Yu Y., Yuan Z., Lin Y., Tan Y., Duan X., Yao H., Wang Y., Liu J. (2023). Radiomic and clinical data integration using machine learning predict the efficacy of anti-PD-1 antibodies-based combinational treatment in advanced breast cancer: A multicentered study. J. Immunother. Cancer.

[19] Christodoulou E., Ma J., Collins G.S., Steyerberg E.W., Verbakel J.Y., Van Calster B. (2019). A systematic review shows no performance benefit of machine learning over logistic regression for clinical prediction models. J. Clin. Epidemiol..

[20] van Griethuysen J.J.M., Fedorov A., Parmar C., Hosny A., Aucoin N., Narayan V., Beets-Tan R.G.H., Fillion-Robin J.C., Pieper S., Aerts H. (2017). Computational Radiomics System to Decode the Radiographic Phenotype. Cancer Res..

[21] Fornacon-Wood I., Mistry H., Ackermann C.J., Blackhall F., McPartlin A., Faivre-Finn C., Price G.J., O’Connor J.P.B. (2020). Reliability and prognostic value of radiomic features are highly dependent on choice of feature extraction platform. Eur. Radiol..

[22] Inkinen S., Liukkonen J., Ylarinne J.H., Puhakka P.H., Lammi M.J., Viren T., Jurvelin J.S., Toyras J. (2014). Collagen and chondrocyte concentrations control ultrasound scattering in agarose scaffolds. Ultrasound Med. Biol..

[23] Cherin E., Saied A., Laugier P., Netter P., Berger G. (1998). Evaluation of acoustical parameter sensitivity to age-related and osteoarthritic changes in articular cartilage using 50-MHz ultrasound. Ultrasound Med. Biol..

